# Concordance in detection of microsatellite instability by PCR and NGS in routinely processed tumor specimens of several cancer types

**DOI:** 10.1002/cam4.6293

**Published:** 2023-06-28

**Authors:** Stephan Bartels, Isabel Grote, Madeleine Wagner, Jannik Boog, Elisa Schipper, Tanja Reineke‐Plaass, Hans Kreipe, Ulrich Lehmann

**Affiliations:** ^1^ Institute of Pathology, Hannover Medical School Hannover Germany

**Keywords:** dMMR, MSI, MSI‐NGS, pentaplex‐PCR

## Abstract

**Background:**

Microsatellite instability (MSI) occurs in several cancer types and is commonly used for prognosis and as a predictive biomarker for immune checkpoint therapy.

**Methods:**

We analyzed *n* = 263 formalin‐fixed paraffin‐embedded (FFPE) tumor specimens (127 colorectal cancer (CRC), 55 endometrial cancer (EC), 33 stomach adenocarcinoma (STAD), and 48 solid tumor specimens of other tumor types) with a capillary electrophoresis based multiplex monomorphic marker MSI‐PCR panel and an amplicon‐based NGS assay for microsatellite instability (MSI+). In total, *n* = 103 (39.2%) cases with a known defect of the DNA mismatch repair system (dMMR), determined by a loss in protein expression of MSH2/MSH6 (*n* = 48, 46.6%) or MLH1/PMS2 (*n* = 55, 53.4%), were selected. Cases with an isolated loss of MSH6 or PMS2 were excluded.

**Results:**

The overall sensitivity and specificity of the NGS assay in comparison with the MSI‐PCR were 92.2% and 98.8%. With CRC cases a nearly optimal concordance was reached (sensitivity 98.1% and specificity 100.0%). Whereas EC cases only show a sensitivity of 88.6% and a specificity of 95.2%, caused by several cases with instability in less than five monomorphic markers, which could be difficult to analyze by NGS (subtle MSI+ phenotype).

**Conclusions:**

MSI analysis of FFPE DNA by NGS is feasible and the results show a high concordance in comparison with the monomorphic marker MSI‐PCR. However, cases with a subtle MSI+ phenotype, most frequently manifest in EC, have a risk of a false‐negative result by NGS and should be preferentially analyzed by capillary electrophoresis.

## Graphical abstract

Microsatellite instability (MSI) analysis of formalin‐fixed paraffin‐embedded (FFPE) DNA by NGS is feasible and the results show a high concordance in comparison with the monomorphic marker MSI‐PCR (overall sensitivity and specificity of the NGS assay in comparison with the MMR‐IHC/MSI‐PCR were 92.2% and 98.8%). However, cases with a subtle MSI+ phenotype, most frequently manifest in endometrial cancer (EC), have a risk of a false‐negative result by NGS and should be preferentially analyzed by capillary electrophoresis.

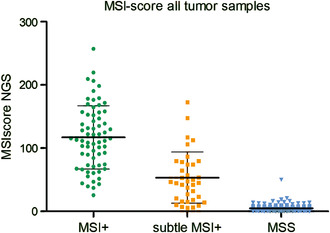

## 1 INTRODUCTION

Microsatellite instability (MSI+) is the result of a defective DNA mismatch repair system (dMMR) arising from either a somatic or a germline alteration in a DNA mismatch repair gene.^1^ Furthermore, epigenetic mechanisms can lead to a MSI+ phenotype, especially promotor methylation of the *MLH1* gene.^2^ MSI+ can be found across many types of cancer with a variable prevalence. Most commonly, endometrial cancer (EC), stomach adenocarcinoma (STAD), and colorectal cancer (CRC) are affected from MSI+ with frequencies of 25%–30%, 15%–20% and ~20%, respectively.^1^, ^3^, ^4^ CRC cases with MSI+ phenotype are predicted to respond to immune checkpoint inhibition against PD1 or PD‐L1 and several studies have shown the efficacy of a pembrolizumab treatment in MSI+ CRC.^5^, ^6^ In addition, anti‐PD1 antibodies can be combined with anti‐CTLA‐4 antibodies, with a robust and durable clinical benefit even as fist‐line treatment in MSI+ CRC.^7^ In STAD MSI+ is mutually exclusive with *ERBB2* amplification and is a positive predictive marker for pembrolizumab plus chemotherapy treatment.^8^, ^9^ Furthermore, beside PD‐L1 expression, MSI+ is a biomarker for the response of immunotherapy‐based treatment of other digestive system tumors.^10^ For EC, MSI+ cases represent an own molecular phenotype.^11^ Currently, different studies investigate a potential predictive role of the MSI+ phenotype for immune checkpoint inhibition in EC.^4^

A current meta‐analysis confirmed that CRC patients with MSI+ have a better prognosis compared to microsatellite stable (MSS) CRC, whereas MSI+ has no prognostic value in EC patients.^12^ This underlines the need of tissue‐specific analysis for prognosis and immunotherapy use.

Defective mismatch repair can be detected by an immunohistochemical (IHC) loss of the MMR heterodimers MSH2/MSH6 and MLH1/PMS2. However, heterogeneous staining patterns and false‐positive or false‐negative staining results can be due to technical pitfalls or caused by non‐truncating mutations in the MMR genes which lead to a nonfunctional but stainable full‐length protein.^13^, ^14^ Therefore, molecular testing is needed in addition to IHC. Two MSI‐PCR approaches widely used are the Bethesda Panel and the Pentaplex panel.^15^, ^16^ The Bethesda Panel consists of two mononucleotide markers (BAT‐25 and BAT‐26) and three dinucleotide markers (D2S123, D5S346, and D17S250) originally developed for the analysis of colorectal cancer specimens in the context of Lynch syndrome, whereas the Pentaplex panel includes five mononucleotide markers (BAT‐25, BAT‐26, NR‐21, NR‐24, and NR‐27).

By using NGS it is possible to analyze a greater number of microsatellite loci in parallel, together with gene mutations, CNV or tumor mutational burden (TMB). In silico thousands of microsatellite loci can be analyzed in exome or genome sequencing datasets,^1^ as well as from targeted panel gene sequencing.^17^ This promises a more sensitive detection of MSI+ than the Bethesda or the Pentaplex panel with only five markers analyzed each. Especially in cases with an uncertain IHC MMR staining pattern a NGS MSI panel might be a possibility to raise the diagnostic precision. To address this question we performed NGS MSI panel analysis and MSI‐PCR with a monomorphic marker assay in 263 tumor samples with known IHC dMMR status (*n* = 103 samples with a loss in MSH2/MSH6 or MLH1/PMS2 protein expression).

## 2 MATERIALS AND METHODS

All tumor samples (*n* = 263) had been taken as part of standard clinical and surgical care between 2018 and June 2022. Retrospective analyses of anonymized diagnostic left over material have been approved by the local ethics committee (Hannover Medical School, Germany, Head: Prof. Schmidt).

Retrospective analysis included *n* = 127 colorectal cancer (CRC), *n* = 55 EC, and *n* = 33 stomach adenocarcinoma specimens (STAD). Additionally, we included *n* = 48 tumors of different origins (*n* = 16 ovarian cancer, *n* = 12 pancreatic cancer, *n* = 8 cholangiocarcinoma, *n* = 7 prostate cancer, and *n* = 5 urothelial carcinoma specimens).

Tumor specimens were cut in 3 μm (for HE staining) and 6 μm thick sections (for manually microdissection). On the HE stains, tumor and corresponding normal tissue were marked by an experienced broad certified pathologist and these regions were scratched with a micro blade from serial unstained sections. DNA isolation was performed with the Maxwell© RSC DNA FFPE kit on a Maxwell© RSC instrument (Promega, Madison). Measurement of the DNA concentration was performed with dsDNA high sensitivity kit on a Qubit™ 2.0 fluorimeter (Invitrogen).

Immunohistochemistry of MSH2, MSH6, MLH1, and PMS2 were performed for every tumor sample on a Benchmark Ultra automated stainer (Ventana). We used monoclonal RTU antibodies for MLH1 (clone M1, mouse), MSH2 (clone G219‐1129, mouse), MSH6 (clone SP63, rabbit), and PMS2 (clone A16‐4, mouse) as recommended by the manufacturer (Roche). Cases with an isolated loss of MSH6 or PMS2 were excluded from further analysis.

### 2.1 Monomorphic marker assay

Tumor and normal tissue DNA were quantified with a qPCR assay. In brief, a 67 bp. fragment of the *APP* gene (fwd: 5′‐TCA GGT TGA CGC CGC TGT‐3′ and rev: 5′‐TTC GTA GCC GTT CTG CTG C‐3′, taqman probe: 5′‐FAM‐ACC CCA GAG GAG CGC CAC CTG‐TAMRA‐3′) was amplified (95°C for 20 s denaturation followed by 40 cycles of 95°C for 3 s an 60°C for 20 s) on a 7500 Fast qPCR system (Applied Biosystems). Depending on the C_T_‐Value of the qPCR assay the DNA was diluted for the PCR (details are available upon request). The PCR for five monomorphic markers (Primer in Table 1) was performed in two Multiplex‐PCR reactions with following conditions: 95°C denaturation for 5 min, 40 cycles of 95°C for 30 s, 55°C for 45 s, and 72°C for 30 s, and an end‐elongation step at 72°C for 5 min. Capillary electrophoresis was carried out on a Beckman CEQ 800 instrument (Beckman Coulter). Analysis of monomorphic markers was carried out by visual inspection of experienced molecular pathologists comparing the patterns in tumor and adjacent normal tissue DNA.

**TABLE 1:** Primer for Multiplex‐PCR reaction of Mix A (NR‐27, BAT‐26, and BAT‐25) and Mix B (NR‐21, and NR‐24).

Primer name	Sequence	Expected length	Reference
***Mix A***			
NR‐27 fwd	5′‐AAC CAT GCT TGC AAA CCA CT‐3′	~88 bp	15, 18
NR‐27 rev‐Cy5	5′‐Cy5‐CGATAATACTAGCAATGACC‐3′		
BAT‐26‐fwd‐Cy5	5′‐Cy5‐TGA CTA CTT TTG ACT TCA GCC‐3′	~118 bp	^19^
BAT‐26‐rev	5′‐AAC CAT TCA ACA TTT TTA ACC C‐3′		
BAT‐25 fwd‐2	5′‐TAC CAG GTG GCA AAG GGC A‐3′	~148 bp	15, 18
BAT‐25 rev‐2‐Cy5	5′‐Cy5‐TCT GCA TTT TAA CTA TGG CTC‐3′		
***Mix B***			
NR21‐fwd	5′‐GAG TCG CTG GCA CAG TTC TA‐3′	~107 bp	15, 18
NR‐21‐rev‐Cy5	5′‐Cy5‐CTG GTC ACT CGC GTT TAC AA‐3′		
NR‐24‐fwd	5′‐GCT GAA TTT TAC CTC CTG AC‐3′	~124 bp	15, 18
NR‐24‐rev‐Cy5	5′‐Cy5‐ATT GTG CCA TTG CAT TCC AA‐3′		

### 2.2 MLH1 promotor methylation assay

Tumor and normal tissue DNA of cases with a loss in MLH1/PMS2 protein expression underwent a bisulfite conversion with the EZ‐Methylation‐Kit (Zymo Research) according to manufacturer's recommendations (total DNA input was 250 ng or maximum amount in 45 μL). PCR with kDNA was performed with following primers: MLH1‐2i5′ (forward) 5′‐TTT TAA AAA YGA ATT AAT AGG AAG AG‐3′ and MLH1‐2i3′‐bio (reverse) 5′‐Biotin‐AAA TAC CAA TCA AAT TTC TCA ACT C‐3′. Conditions of the PCR reaction were 5 min at 95°C; 45 cycles of 30 s at 95°C, 45 s at 55°C and 30 s at 72°C; followed by an end‐elongation for 5 min at 72°C. Pyrosequencing was performed as described^20^ with two assays one behind the other on the same PCR product (pyro primer: MLH1‐pyro1 5′‐AAA YGA ATT AAT AGG AAG AG‐3′ and MLH1‐pyro2 5′‐AAG YGT ATA TTT TAG GTA G‐3) to analyze eight CpG sites in the promotor sequence. The threshold for promotor hypermethylation has been set at 20% mean methylation level in tumor kDNA above the normal tissue kDNA methylation level (normalized by eight conversion control sites in the sequence, four in each sequencing reaction).

### 2.3 Next‐generation sequencing

For NGS analysis we used the MSI community Panel (ThermoFisher scientific) with 15 ng FFPE DNA input per tumor sample and for corresponding normal tissue. The MSI community panel consists of 76 amplicons, including the Bethesda and Pentaplex markers (for a detailed overview Table S1). After manual library preparation with the AmpliSeq 2.0 kit, template preparation was performed on the IonChef system (LifeTechnologies) and 48 samples were loaded on an Ion 540 Chip and sequenced on an Ion GeneStudio™ S5 System (LifeTechnologies) with a minimum of 250.000 reads per sample. Data analysis was performed with the MSICall plug‐in v4.2 on the Torrent Server. As MSS control FFPE DNA from the cell line JVM‐13 was used, if normal tissue was not available.

## 3 RESULTS

**FIGURE 1:**
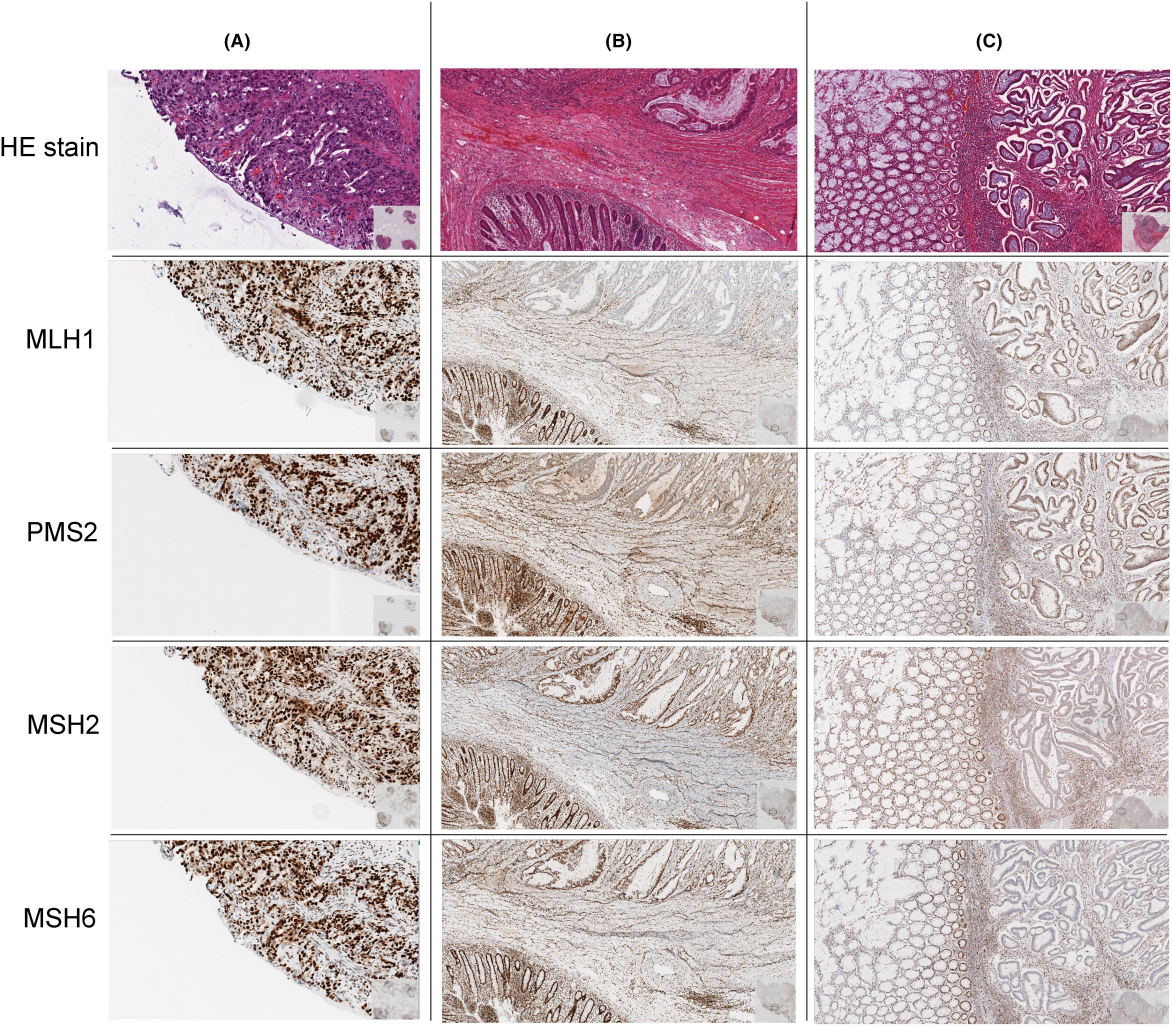
Exemplary cases selected for this study. (A) Infiltration of a CRC MSS with a present expression of MLH1/PMS2 and MSH2/MSH6. (B) Recurrent small intestine carcinoma MSI+ with an immunohistochemically loss of MLH1/PMS2. (C) Infiltration of a CRC MSI+ with an immunohistochemically loss of MSH2/MSH6.

The characteristics of the selected archived tumor samples are listed in Table 2. In total, 103 cases with MSI+ and 160 cases with MSS analyzed in the molecular pathology routine with our monomorphic marker assay including BAT‐25, BAT‐26, NR‐21, NR24, and NR‐27 were selected. The MSI+ status was confirmed by immunohistochemistry by loss of the mismatch repair marker MSH2/MSH6 or MLH1/PMS2 (see Figure 1). In total, 55 out of 103 (53.4%) MSI cases show a loss in MLH1/PMS2 protein expression (Figure 1B). In 49 out of 55 cases we performed a methylation analysis of the MLH1 promotor region and found 32 cases (65.3%) to be hypermethylated which indicates a sporadic tumor development.

**TABLE 2:** Clinical data for all tumor samples under study.

	Cases (%)	Mean age	Gender(female, %)	MSI + (5 markers)	MSI + (2–4 markers)	MSS
All samples	263 (100%)	63.9 (range 16–97)	148 (56.3%)	67	36	160
CRC	127 (48.3%)	63.5 (range 16–89)	62 (48.8%)	41	12	74
EC	55 (20.9%)	62.1 (range 40–89)	55 (100.0%)	16	18	21
GIST	33 (12.5%)	65.0 (range 40–97)	13 (39.4%)	8	2	23
Other	48 (18.3%)	66.5 (range 36–86)	18 (37.5%)	2	4	42

MSI+ can affect all five tested markers (*n* = 67), or only two up to four markers (*n* = 36). This subtle MSI+ phenotype with less than five affected markers is more common in EC than in CRC cases and can be difficult to detect.

**FIGURE 2:**
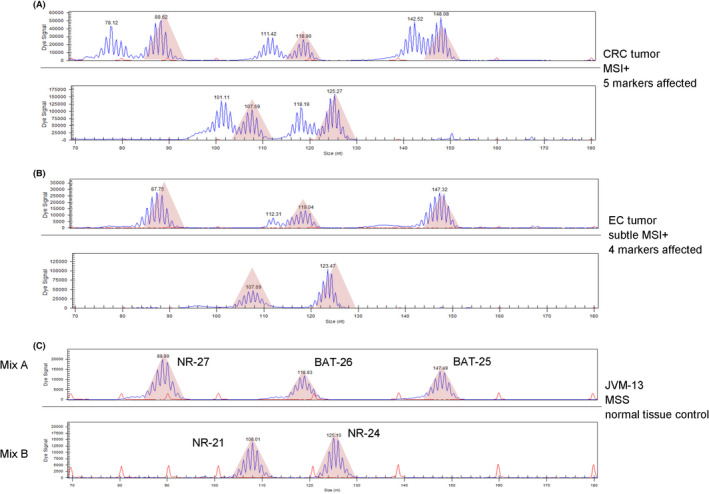
Exemplary results from the capillary electrophoresis with the monomorphic marker assay. (A) CRC MSI+ case with all five markers affected. (B) EC MSI+ case with only four affected markers, NR‐24, NR‐27, BAT‐25, and BAT‐26. (C) Cell line JVM‐13 MSS, serve as MSS control with a Gaussian distribution of the PCR products. The red transparent triangles visualize the microsatellite stable PCR‐product amplified from JVM‐13.

In Figure 2 an exemplary output from the capillary electrophoresis is shown for a MSI+ CRC tumor with five monomorphic markers clearly affected (Figure 2A) and a MSI+ EC tumor with only four markers subtly affected (Figure 2B) by microsatellite instability (BAT‐26, NR‐24, and NR‐27). The MSS cell line JVM‐13 (Figure 2C) served as a normal tissue control and shows a nearly Gaussian distribution of the PCR product length in the capillary electrophoresis (red transparent triangles).

**FIGURE 3:**
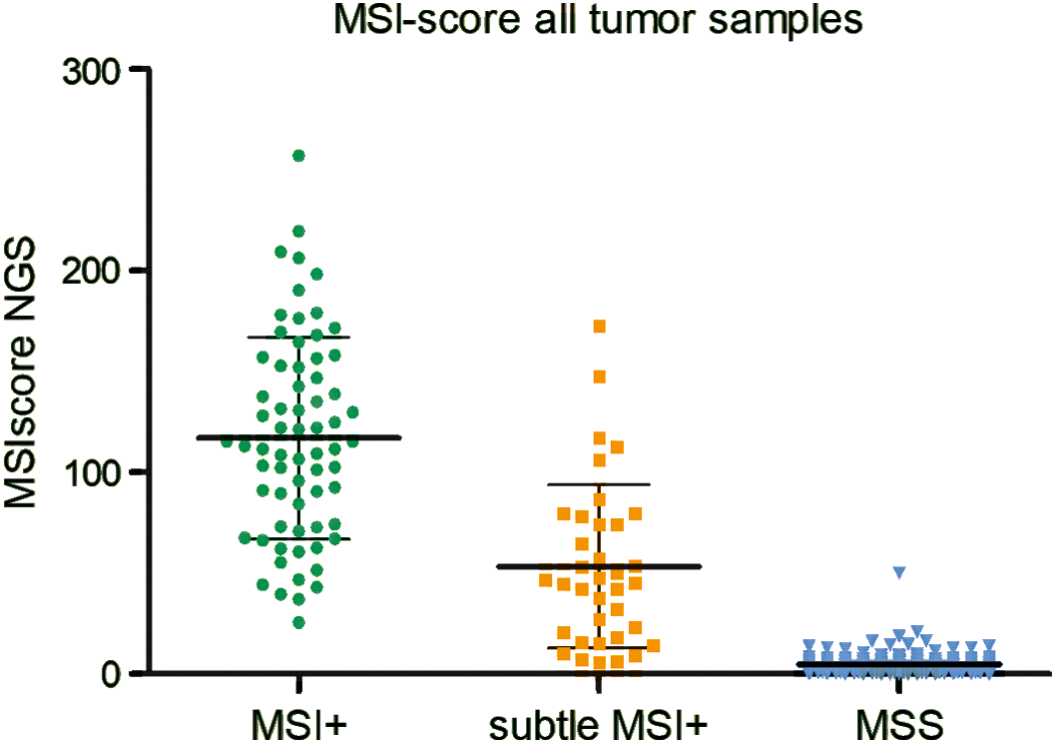
MSI‐scores from the NGS analysis for all tested samples. Mean an SD values are listed in Table 3.

To compare the results of the monomorphic marker assay with the NGS based assay, all samples with available normal tissue were sequenced twice (tumor DNA and normal tissue DNA) with the corresponding normal tissue DNA as selected run control in the MSICall plug‐in. All samples without available normal tissue DNA were analyzed in comparison to JVM‐13 cell line DNA, run as control to calculate the MSI‐score. Calculated MSI‐score mean values for all sample groups are listed in Table 3 and visualized in Figure 3.

**TABLE 3:** Mean MSI‐score thresholds for NGS analysis of all cases as well as for the different tumor entities.

		Monomorphic markers			Mean NGS MSI‐score (SD)			Classified correctly with NGS		
	Total samples	MSI + 5 marker	Subtle MSI + 2–4 marker	MSS	MSI + 5 marker	Subtle MSI + 2–4 marker	MSS	MSI + 5 marker	Subtle MSI + 2–4 marker	MSS
All samples	263	67	36	160	117.1 (50.1)	53.6 (40.4)	4.7 (5.4)	67 (100.0%)	28 (77.8%)	158 (98.8%)
CRC	127	41	12	74	134.5 (47.5)	90.8 (46.0)	4.3 (3.5)	41 (100.0%)	11 (91.7%)	74 (100.0%)
EC	55	16	18	21	71.6 (33.0)	35.8 (20.2)	4.6 (3.9)	16 (100.0%)	14 (77.8%)	20 (95.2%)
STAD	33	8	2	23	120.3 (33.0)	34.5 (26.4)	6.3 (10.8)	8 (100.0%)	1 (50.0%)	22 (95.7%)
other	48	2	4	42	110.0 (77.2)	31.3 (19.9)	4.6 (4.4)	2 (100.0%)	2 (50.0%)	42 (100.0%)

Abbreviations: SD: standard deviation.

MSI+ tumors with instability in all five monomorphic markers show the highest values of the MSI‐score in the NGS analysis (mean 117.1, range 39.6–257.2). In tumors with subtle MSI+ phenotype the NGS MSI‐score (mean 53.6, range 6.35–173.0) is clearly lower than in the MSI+ cases. The MSS cases have the lowest MSI‐score in the NGS analysis (mean 4.7, range 0.0–50.1). The difference between the groups is highly significant (non‐parametric one‐way ANOVA, *p* < 0.0001).

**FIGURE 4:**
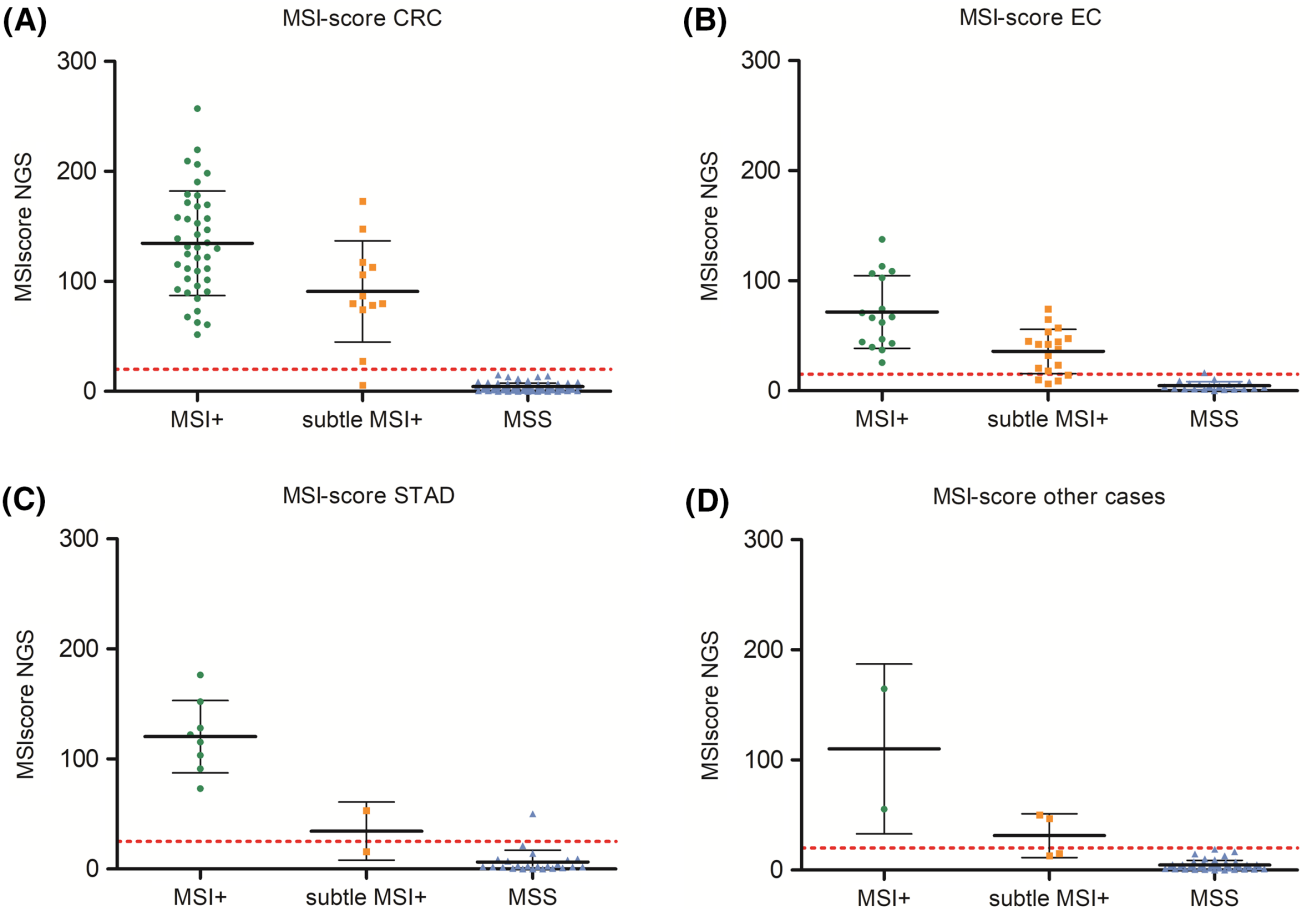
MSI‐scores from the NGS analysis for all tested samples, distinguished in the different tumor sub‐types: CRC (A), EC (B), STAD (C), and other solid tumor cases (D). Mean an SD values are listed in Table 3. The red‐dotted line represents the optimal threshold for NGS MSI status (see Table 4).

Figure 4 shows a detailed overview of the MSI‐score in the different tumor entities (mean and SD values are listed in Table 3). Thereby, it is clearly visible that the mean MSI‐score for CRC (Figure 4A) and EC (Figure 4B) cases differ by almost a factor of two (134.5 for CRC vs. 71.6 for EC). For subtle MSI+ cases the mean MSI‐score is 90.8 for CRC and 35.8 for EC. The STAD tumor and other solid tumors show mean values similar to the CRC tumors in case of MSI+ status. The different mean MSI‐score values make it necessary to determine different thresholds for the NGS MSI analysis to reach an optimal sensitivity and specificity. With NGS MSI thresholds of 15 (EC), 20 (CRC and other solid tumors), and 25 (STAD) all unequivocal MSI+ cases (67/67, 100%) were classified correctly (Table 4). For the subtle MSI+ cases only 28/36 (77.8%) could be classified correctly. From the MSS cases 158/160 (98.8%) could be classified correctly by the NGS analysis.

**TABLE 4:** MSI‐score thresholds for NGS analysis of all cases as well as for the different tumor entities. MSI+ and subtle MSI+ cases are summarized to allow the calculation of a combined overall sensitivity and specificity.

		Monomorphic markers		NGS						
	Total samples	MSI+	MSS	MSI‐score threshold	MSI+	MSS	Sensitivity	Specificity	PPV	NPV
All samples	263	103	160	15–25^a^	97	166	92.2%	98.8%	99.0%	95.2%
CRC	127	53	74	20	52	75	98.1%	100.0%	100.0%	98.7%
EC	55	34	21	15	31	24	88.6%	95.2%	96.9%	83.3%
STAD	33	10	23	25	10	23	90.0%	95.7%	90.0%	95.7%
other	48	6	42	20	4	44	66.6%	100.0%	100.0%	95.5%

Abbreviations: NPV, negative predictive value; PPV, positive predictive value.

^a^ Specific threshold depends on the tumor type of the sample.

For EC, subtle MSI+ cases are at risk to be classified false negative in MSI NGS analysis. Only 14/18 (77.8%) of the subtle MSI+ cases were classified as MSI+ by the NGS approach. Furthermore, one MSS EC case was falsely classified as MSI+ by NGS with a threshold of 15. However, 15 is the optimal threshold for EC in this set. A higher threshold would cause even more false‐negative MSI+ cases.

The sensitivity of the NGS assay in comparison with MMR‐IHC/MSI‐PCR is 92.2% and the specificity is 98.8% (Table 4). For CRC a nearly perfect concordance of both methods could be reached, only one subtle MSI+ sample was classified false‐negative as MSS by NGS. Sensitivity for CRC is 98.1% and specificity 100.0%. For EC, four subtle MSI+ cases were classified false‐negative and one MSS sample was classified false‐positive with the used threshold. This leads to a sensitivity of 88.6% and a specificity of 95.2%. This is comparable with the STAD cases, where one subtle MSI+ sample was classified false‐negative and one MSS sample was classified false‐positive by NGS (sensitivity 90.0% and specificity 95.7%, Table 4).

## 4 DISCUSSION

Several studies are available that tested the accuracy of MSI+ results in different tumor specimens (reviewed in reference 4). Nevertheless, our study has some advantages. First, most of the available studies only compare MMR‐IHC results with MSI‐PCR.^21^, ^22^, ^23^, ^24^, ^25^, ^26^, ^27^ We included cases with consistent dMMR and MSI‐PCR results and compared them with MSI‐NGS. Second, studies which compare IHC, MSI‐PCR and NGS results investigate a smaller number of total cases.^21^, ^22^, ^23^, ^24^ Third, most of the available studies investigated only CRC,^25^, ^26^, ^27^ STAD,^28^, ^29^ or EC^30^, ^31^ and did not compare the results of all three tumor specimens with the same laboratory workflow. In the study of Kang et al.^32^ the Illumina TSO500 panel was used for MSI‐NGS analysis and a comparison with MSI‐PCR and MMR‐IHC was provided. The study included 1942 cases and found 100% concordance. However, despite the large total sample number, only three EC cases were included which show the subtle MSI+ phenotype more often. In Table S2 we summarize the characteristics and investigations of the referred studies.

MSI+ cases with all five monomorphic markers affected in the MSI‐PCR display a perfect concordance with the NGS‐derived MSI+ results (67/67, 100.0%). However, from the cases with a subtle MSI+ phenotype with only two to four (out of five) markers affected in the MSI‐PCR, only 28/36 (77.8%) were classified correctly as MSI+ by the NGS assay. The NGS assay covers 76 MSI‐markers, including the Bethesda and the Pentaplex markers. It might be conceivable that some of these have such a high variability in the sequencing results that MSS cases become false‐positive and subtle MSI+ cases become false negative. The basic settings of the MSIcall plug‐in are MSIscore <50 for MSS, equivocal for 50 to 60, and MSIscore >60 for MSI. These values applied to our dataset would cause a perfect specificity without false‐positive cases, but almost half of the PCR‐MSI+ EC cases would be classified false‐negative by NGS. Therefore, it is necessary to evaluate lab‐ and tissue‐specific thresholds of the MSIcall plug‐in to reach optimal sensitivity and specificity. Another reason for false‐positive cases could be that some individuals show bi‐phasic distribution of single MSI markers in all cells. This could only be interpreted correctly with available normal tissue for comparison.

Data evaluation on the Torrent Server with the MSIcall plug‐in is very easy but time consuming; the plug‐in analysis needs approximately 2 h and must be started anew for every sample with normal tissue separately and then analyses all samples against a single reference. This could be solved by analyzing all tumor samples against a MSS cell line control without sequencing available patient normal tissue. However, patient specific normal tissue as reference optimized both sensitivity and specificity and should be included in the analysis if possible. In addition, the MSIcall plug‐in provides no opportunity to visualize the sequencing reads for each individual marker, which would be a helpful tool for visually inspection.

MSI analysis employing NGS allows testing several different molecular markers in a single analysis (SNV, indels, TMB, CNV, HRD) with a comprehensive targeted panel or whole exome sequencing. The commercially available OCA+ panel from ThermoFisher covering 500 tumor relevant genes, TMB, CNV, HRD, and MSI includes exactly the same MSI marker panel presented in our dataset. Therefore, the MSI performance of this assay should be very similar to the results presented here. Large NGS panels with hundreds of genes like TSO500 or OCA+ are relatively expensive and have a long turn‐around time in the laboratory, especially a complex data evaluation. For many solid tumor cases a small defined set of genetic biomarker plus MSI‐testing is a cost‐effective and therapeutically useful approach.

In conclusion, MSI‐NGS analysis with FFPE tumor tissue is feasible and shows a high concordance in comparison with MMR‐IHC and MSI‐PCR. However, EC specimens show a lower MSI‐score in general, especially in cases with a subtle MSI+ phenotype and have a high risk of false‐negative results. In cases of immunohistochemically dMMR EC with a discordant MSS NGS result validation of the NGS result with a MSI‐PCR analysis is strongly recommended.

## AUTHOR CONTRIBUTIONS

**Stephan Bartels:** Conceptualization (lead); data curation (lead); formal analysis (lead); investigation (lead); project administration (lead); writing – original draft (lead). **Isabel Grote:** Data curation (supporting); formal analysis (supporting); writing – review and editing (supporting). **Madeleine Wagner:** Data curation (supporting); formal analysis (supporting). **Jannik Boog:** Data curation (supporting); formal analysis (supporting). **Elisa Schipper:** Data curation (supporting); formal analysis (supporting). **Tanja Reinecke‐Plaass:** Conceptualization (supporting); writing – review and editing (supporting). **Hans Kreipe:** Conceptualization (supporting); resources (lead); writing – review and editing (supporting). **Ulrich Lehmann:** Conceptualization (supporting); writing – original draft (supporting); writing – review and editing (lead).

## CONFLICT OF INTEREST STATEMENT

All authors declare that no conflict of interest exists.

## ETHICS STATEMENT

Retrospective analyses of anonymized diagnostic left over material have been approved by the local ethics committee (Hannover Medical School, Germany, Head: Prof. Schmidt).

## Supporting information


Table S1:

Table S2:

## Data Availability

Data generated or analyzed during this study are included in this published article.
